# Leisure-time, occupational, and commuting physical activity and risk of type 2 diabetes in Japanese workers: a cohort study

**DOI:** 10.1186/s12889-015-2362-5

**Published:** 2015-10-02

**Authors:** Toru Honda, Keisuke Kuwahara, Tohru Nakagawa, Shuichiro Yamamoto, Takeshi Hayashi, Tetsuya Mizoue

**Affiliations:** Hitachi Health Care Center, Hitachi, Ltd., 4-3-16 Ose-cho, Hitachi Ibaraki, Japan; Department of Epidemiology and Prevention, Center for Clinical Sciences, National Center for Global Health and Medicine, 1-21-1 Toyama, Shinjuku-ku, Tokyo, Japan; Teikyo University Graduate School of Public Health, 2-11-1 Kaga, Itabashi-ku, Tokyo, Japan

**Keywords:** Intensity of exercise, Dose–response relationship, Prevention, Type 2 diabetes, Cohort studies, Asians

## Abstract

**Background:**

Physical activity has been suggested to reduce the risk of type 2 diabetes. However, evidence is limited regarding whether vigorous-intensity activity yields the same benefits in preventing type 2 diabetes compared with an equivalent dose of moderate-intensity activity as well as other type of physical activity. We examined the risk of type 2 diabetes associated with exercise intensity during leisure and occupational and commuting physical activity among Japanese individuals.

**Methods:**

Participants included 26,628 workers (23,207 men and 3,421 women) aged 30 to 64 years without diabetes at baseline. There was 6 years of follow-up maximum. Leisure-time exercise, occupational physical activity, and duration of walking to and from work were self-reported. Diabetes was diagnosed by using HbA_1c_, fasting or random blood glucose, and self-report. We used Cox regression analysis to estimate the hazard ratio (HR) and the 95 % confidence interval (CI) of incident diabetes.

**Results:**

During a mean follow-up of 5.2 years, 1,770 participants developed type 2 diabetes. Compared with individuals who engaged in no exercise, the HRs (95 % CIs) for <7.5, 7.5 to <15.0, and ≥15.0 MET-hours per week of exercise were 0.94 (0.81, 1.08), 1.07 (0.88, 1.30), and 0.90 (0.67, 1.21), respectively, among individuals who engaged in moderate-intensity exercise alone; 0.68 (0.44, 1.06), 0.86 (0.54, 1.34), and 0.89 (0.56, 1.41), respectively, among individuals who engaged in vigorous-intensity exercise alone; and 0.70 (0.44, 1.11), 0.57 (0.37, 0.90), and 0.76 (0.52, 1.11), respectively, among individuals who engaged in the two intensities, with adjustments for potential confounders and the total volume of exercise. Occupational physical activity and walking to and from work were not associated with diabetes.

**Conclusions:**

The results suggest that vigorous-intensity exercise can reduce the risk of type 2 diabetes among Japanese workers.

**Electronic supplementary material:**

The online version of this article (doi:10.1186/s12889-015-2362-5) contains supplementary material, which is available to authorized users.

## Backgrounds

Diabetes, an established risk factor for vascular disease [1, 2], causes macrovascular and microvascular complications [3]. The number of patients with diabetes worldwide reached 382 million in 2013 [4] and is projected to keep increasing [4]. Japan is in the top 10 countries with the largest number of diabetic patients worldwide [4], and a recent national survey [5] shows that approximately 9.5 million individuals have suspected diabetes in Japan. This metabolic disease causes an economic burden. The cost due to diabetes in Japan has been increasing and reached more than one trillion yen in 2011 [6]. Physical activity has been considered to be a key strategy for the prevention of type 2 diabetes. Based on epidemiological evidence mainly on leisure-time physical activity for health, including diabetes prevention, the US government [7] and the World Health Organization [8] recommend adults engage in at least 150 min per week of moderate-intensity physical activity, 75 min per week of vigorous-intensity physical activity, or an equivalent combination of the two intensities.

Nonetheless, some important issues need to be addressed. First, a recent meta-analysis of several cohort studies [9] suggested that vigorous-intensity physical activity is associated with a greater reduction of diabetes risk compared with moderate-intensity physical activity. However, the most of the studies included in that review [10–17] were limited because individuals who engaged in moderate-intensity physical activity might also engage in vigorous-intensity physical activity and vice versa [10–17], resulting in mixed results. Second, some studies suggested that a higher intensity of physical activity can produce greater disease risk reduction at even a low-level of physical activity above the sedentary level [7]. In a Taiwanese cohort study, vigorous-intensity exercise below the recommended dose showed a greater reduction in mortality rates than the same dose of moderate-intensity exercise [18]. Few studies [13, 16], however, examined the diabetes risk below the recommended dose and the intensity of activity. Third, data on diabetes risk are limited in relation to specific type of leisure-time activity and other domain of physical activity (i.e., occupational and commuting physical activity) [9]. Finally, most data cited in the above physical activity guidelines were generated with Caucasian study participants [7]. Asian cohort studies reporting the association of physical activity with diabetes risk [19–23] have had limited assessments regarding the dose or intensity of physical activity. To overcome these issues, we examined the associations between leisure-time, occupational, and commuting physical activity and the risk of type 2 diabetes in a Japanese working population with a special emphasis on the intensity and dose for leisure-time physical activity.

## Methods

### Study procedure

The present study was conducted as a sub-study of the Japan Epidemiology Collaboration on Occupational Health (J-ECOH) Study, an ongoing, large-scale study among workers in several companies [24, 25]. The J-ECOH Study was announced in each company by using posters, and workers were given an opportunity to refuse the use of their data for research, according to the Japanese Ethical Guidelines for Epidemiological Research. The study protocol was approved by the Ethics Committee of the National Center for Global Health and Medicine, Japan.

Of the participating companies in the J-ECOH Study, the present analysis included data from one company (an electrical machinery and apparatus manufacturing) where detail information on physical activity has been collected as a part of periodic health check-ups since 2006.

### Participants

In Japan, workers are obliged to undergo health checkup at least once a year under the Industrial Safety and Health Act. A total of 40,948 workers (34,700 men and 6,248 women) aged 30 to 64 years received health check-ups between April 2006 and March 2007 (baseline period). Of these individuals, 11,434 workers were excluded due to lack of information on the variables needed to diagnose diabetes (*n* = 7,655), having a history of diabetes (defined using HbA_1c_, fasting or random plasma glucose, a medical history of diabetes, or currently taking medication for diabetes) (*n* = 3,376). Workers were also excluded if they having a history of cancer, cardiovascular disease, or stroke (*n* = 766). We excluded an additional 2,964 workers due to lack of information on exposure or covariates (*n* = 2,515) as well as engagement in only unspecified leisure activity (named “Other”) (*n* = 487). Some of the participants met one or more of the exclusion criteria. Finally, we excluded 1,303 participants who did not attend any subsequent health check-up or who did not have data on HbA_1c_ or blood glucose in a subsequent health examination, which left 26,628 workers (23,207 men and 3,421 women) aged 30 to 64 years for inclusion in our study.

### General health examination

Body height was measured to the nearest 0.1 cm and body weight to the nearest 0.1 kg. BMI was calculated as weight in kilograms divided by squared height in meters. The systolic and diastolic blood pressures were assessed using an oscillometric method with an automated sphygmomanometer (BP-203RV III; Colin, Tokyo, Japan) in a sitting position after a 5 min rest. The history of disease, work-related factors, and health-related lifestyle factors, including physical activity, alcohol consumption, and sleep duration, were collected using a standard questionnaire. Biochemical measurements included plasma glucose and HbA_1c_. The blood glucose levels were determined using the glucose electrode technique. HbA_1c_ was measured with an HPLC method. HbA_1c_ was assessed according to a standard method used by the Japan Diabetes Society. We converted the HbA_1c_ measurements to the National Glycohemoglobin Standardization Program equivalent value (%) using the formula: ${\mathrm{HbA}}_{1\mathrm{c}}\left(\%\right)=1.02\times {\mathrm{HbA}}_{1\mathrm{c}}\left(\mathrm{Japan}\ \mathrm{Diabetes}\ \mathrm{Society}\right)\left(\%\right)+0.25\%$ [26].

### Physical activity questionnaire

Participants were asked if they were regularly engaging in any physical activity during their leisure time. If they engaged in physical activity, they were also asked to choose up to 3 activities among a list of 20 activities and describe the frequency (times per month) as well as the duration (minutes) for each activity. If participants were engaged in activities that were not listed in the questionnaire, they were instructed to choose an activity of similar intensity from the list.

Of the 20 regular exercise or sports activities, one activity, “Other,” was not used for further analysis. The value of metabolic equivalents (METs) for each activity was assigned according to a compendium of physical activities [27]. If the MET value of an activity was not listed in the compendium, we assigned a MET value from a similar activity. Of the 19 activities, 13 (walking not for work or commuting, walking fast not for work or commuting, swimming, golf practice, golf, baseball, softball, bike cycling, table tennis, pang pong, badminton, muscle strength training, and radio gymnastics) were classified as moderate activities (3 to 6 METs) and 6 (light jogging [approximately 6 min/km], jogging, soccer, tennis, aerobics, and jump rope) were classified as vigorous activities (>6 METs), and these activities were used to calculate the weekly MET-hours of leisure-time exercise by multiplying the METs, duration, and frequency of the activity. These activities covered the most of common activities in Japan [28]. Following the physical activity guidelines, the participants were categorized into four groups according to the dose of leisure-time exercise per week: inactive (0 MET-hours), low (0.1 to <7.5 MET-hours), medium (7.5 to <15.0 MET-hours), and high (≥15.0 MET-hours) [7, 8]. These cutoff points were used in previous studies [18, 29]. Individuals in each category of leisure-time exercise dose were further divided into the following three groups: individuals who engaged in moderate-intensity exercise alone, vigorous-intensity exercise alone, and both moderate- and vigorous-intensity exercise.

Occupational physical activity was assessed by a single question with four response options (mostly sedentary, mostly standing, walking often, or fairly active). Duration of walking for commuting to and from work (min per day) was self-reported.

### Assessment of other variables

The information on smoking, alcohol consumption, sleep duration, shift work, and a family history of diabetes was collected using a standard questionnaire during the health check-ups. Smoking status (never, past, or current) and, if the individual was a smoker, the number of cigarettes smoked per day were also ascertained during the health check-ups. The total amount of alcohol consumption for each individual was calculated using the data on the frequency (number of days per week) and amount of consumption of common alcoholic beverages (Japanese sake, beer, whiskey, *shochu*, *chuhai*, and wine) per day, which was indicated by an equivalent amount of one unit (go) of Japanese sake. One go of Japanese sake contains approximately 23 g of ethanol.

### Assessment of type 2 diabetes

Diabetes was identified using data from the annual health check-ups for a maximum of 6 years after the baseline examination. Diabetes was defined as HbA_1c_ ≥6.5 % (48 mmol/mol), fasting plasma glucose ≥126 mg/dl (7.0 mmol/l), random plasma glucose ≥200 mg/dl (11.1 mmol/l), or currently under medical treatment for diabetes. Individuals without diabetes at baseline who met any of the above conditions in the subsequent check-ups were considered to have an incident case of type 2 diabetes.

### Statistical analysis

The descriptive results of study population are expressed as the mean for continuous variables and percentages for categorical variables. The differences across the dose of leisure-time physical activity were tested by using linear regression for continuous variables and logistic regression for categorical variables.

Person-time was calculated from the date of the baseline examination to the date of diagnosis with diabetes at a subsequent examination or to the date of the last examination, whichever came first. The HR and the 95 % CI for the incidence of diabetes associated with leisure-time exercise, occupational physical activity, and walking to and from work were estimated by using Cox proportional hazards models. First, we adjusted for age (years, continuous) and sex (model 1). Then, model 2 was further adjusted for shift work (yes or no), smoking status (non-smoker, current smoker consuming 1 to 10, 11–20, or ≥21 cigarettes per day), alcohol consumption (non-drinker, drinker consuming <1, 1 to <2, ≥2 go of Japanese sake equivalent per day [1 go of Japanese sake contains approximately 23 g of ethanol]), sleep duration (<5, 5 to <6, 6 to <7, or ≥7 h per day), hypertension (yes or no, defined as a systolic blood pressure ≥140 mmHg, a diastolic blood pressure ≥90 mmHg, or currently taking medication for hypertension), a family history of diabetes (yes or no), and other two types of physical activity. That is, occupational physical activity (mostly sedentary, mostly standing, walking often, or fairly active) and commuting physical activity (<20, 20 to <40, or ≥40 min of walking for commuting to and from work) were adjusted for leisure-time exercise, leisure-time exercise and commuting physical activity were adjusted for occupational physical activity, and leisure-time exercise and occupational physical activity were adjusted for commuting physical activity. In model 3, BMI (kg/m^2^, continuous) was adjusted for.

Trends in the association between physical activity and diabetes risk were assessed by determining the median value in each category of leisure-time physical activity. The lowest category of each activity was considered as a reference. For combined associations of the dose and intensity of leisure-time physical activity, participants who did not engage in leisure-time exercise were references for our analyses. We repeated the analysis for dose of moderate-intensity exercise and vigorous-intensity exercise, respectively. In addition, we conducted analysis for each specific type of leisure-time exercise and risk of diabetes; participants were classified as those who engaged in specific activity or not. We tested the proportional-hazards assumption with the Schoenfeld residuals. We found no significant deviations for all of the covariates, except for smoking and occupational physical activity. As a sensitivity analysis, we excluded participants with a follow-up period of less than three years. Two-sided *P* values <0.05 were considered to be statistically significant. All analyses were performed with Stata version 13.1 (Stata Corp, College Station, Texas).

## Results

In the present study, the participants who remained main analysis were less likely to include women (12.9 % vs. 22.5 %) and to have family history of diabetes (13.9 % vs. 17.4 %) and they had a lower HbA1c level compared with the excluded participants (*n* = 15,701). Included participants were more likely to engage in shift work (18.8 % vs. 13.3 %) and walk for commuting and worked longer hours (monthly overtime work of ≥45 h: 36.5 % vs. 26.6 %). Other variables including age, BMI, hypertension, smoking, alcohol use, sleep duration, occupational physical activity were not materially different between the excluded and included participants. Overall, the mean age of was 45.3 years (standard deviation: 8.4) and the BMI was 23.4 (standard deviation: 3.2) among the participants who remained analysis. Table 1 shows the characteristics of the participants according to the dose of leisure-time exercise. Compared with workers who engaged in a lower dose of leisure-time exercise, those who engaged in a higher dose of leisure-time exercise were older, had a higher BMI, tended to engage in sedentary work, and slept more, but were less likely to be a female, a shift worker or a smoker and walked less during commuting. Other factors, including alcohol use, having hypertension, and a family history of diabetes, were not materially different across the category of leisure-time exercise (*P* for trend >0.1).

**Table 1:** Baseline characteristics according to the leisure-time exercise dose

	Inactive	Low dose	Physical activity meeting recommendation^a^		*P* for trend^b^
			Medium dose	High dose	
*n*	17,437 (65.5)	4,331 (16.3)	2,537 (9.5)	2,323 (8.7)	
Male, %	14,905 (85.5)	3,886 (89.7)	2,281 (89.9)	2,135 (91.9)	<0.001
Age, years	45.2 ± 8.2	44.6 ± 8.7	45.8 ± 8.5	46.4 ± 8.8	<0.001
BMI, kg/m^2^	23.3 ± 3.2	23.4 ± 3.2	23.7 ± 3.0	23.5 ± 2.9	<0.001
BMI ≥25 kg/m^2^	4,727 (27.1)	1,173 (27.1)	752 (29.6)	608 (26.2)	0.90
Shift work	3,329 (19.1)	874 (20.2)	415 (16.3)	379 (16.3)	<0.001
Walking <20 min to and from work	9,299 (53.3)	2,374 (54.8)	1,402 (55.3)	1,308 (56.3)	0.002
Sedentary work	10,311 (59.1)	2,574 (59.4)	1,632 (64.3)	1,413 (60.8)	0.001
Exercise dose, weekly MET-hr	0	3.7 (2.2, 5.4)	10.5 (9.0, 12.6)	23.0 (18.2, 32.9)	
Sleeping <6 hrs per day	9,224 (52.9)	2,021 (46.7)	1,214 (47.9)	1,052 (45.3)	<0.001
Current drinker^c^	1,764 (10.1)	370 (8.5)	250 (9.9)	255 (11.0)	0.31
Current smoker	7,750 (44.5)	1,751 (40.4)	945 (37.3)	829 (35.7)	<0.001
Hypertension	3,092 (17.7)	706 (16.3)	486 (19.2)	411 (17.7)	0.55
Family history of diabetes	2,417 (13.9)	570 (13.2)	362 (14.3)	339 (14.6)	0.29

Data are shown in mean ± standard deviation for continuous variables, median (interquartile range) for exercise dose, and *n* (%) for categorical variables

^a^≥7.5 MET-hr per week

^b^ *P* for the trend was calculated using linear regression for continuous variables and logistic regression for categorical variables

^c^Consuming ≥2 go of Japanese sake equivalent per day (1 go of Japanese sake contains approximately 23 g of ethanol)

During a mean follow-up of 5.2 years with 139,739 person-years, 1,770 workers developed type 2 diabetes. As shown in Table 2, there was a significant inverse association between the dose of leisure-time exercise and the risk of type 2 diabetes in the age- and sex-adjusted model. The HRs (95 % CIs) were 1.00 (reference), 0.83 (0.73, 0.95), 0.89 (0.76, 1.05), and 0.78 (0.65, 0.93) for increasing doses of leisure-time exercise (*P* for trend = 0.003). Further adjustment for other potential confounders, including physical activities during work and commuting as well as BMI, slightly attenuated the association (*P* for trend = 0.024). The corresponding HRs (95 % CIs) were 1.00 (reference), 0.87 (0.76, 1.00), 0.92 (0.78, 1.08), and 0.83 (0.69, 0.99).

**Table 2:** Hazard ratios (95 % confidence intervals) of incident type 2 diabetes according to the dose of leisure-time moderate- and vigorous-intensity exercise

	Inactive	Low dose	Physical activity meeting recommendation		*P* for trend^a^
			Medium dose	High dose	
Medium exercise dose	0	3.7	10.5	23.0	
No. of subjects	17,437	4,331	2,831	2,029	
No. of cases	1,210	257	166	137	
Person-years	91,266	23,080	13,278	12,116	
Cases/ 10,000 person-years	133	111	125	113	
Model 1^b^	1.00 (reference)	0.83 (0.73, 0.95)	0.89 (0.76, 1.05)	0.78 (0.65, 0.93)	0.003
Model 2^c^	1.00 (reference)	0.89 (0.78, 1.02)	0.95 (0.80, 1.11)	0.85 (0.71, 1.01)	0.06
Model 3^d^	1.00 (reference)	0.87 (0.76, 1.00)	0.92 (0.78, 1.08)	0.83 (0.69, 0.99)	0.024

^a^ *P* for the trend was calculated using Cox proportional hazard regression, and ordinal numbers 0, 4, 11, and 23 were assigned to increasing levels of leisure-time physical activity, which was treated as continuous variable

^b^Adjusted for age (years, continuous) and sex

^c^Adjusted for age (years, continuous), sex, shift work (yes or no), sleep duration (<5, <6, 6 to <7, or ≥7 h per day), alcohol consumption (non-drinker, current drinker consuming <1, 1 to <2, or ≥2 go of Japanese sake equivalent per day [1 go of Japanese sake contains approximately 23 g of ethanol]), smoking (never, past, current smoker consuming 1 to 20 or ≥21 cigarettes per day), hypertension (yes or no), a family history of diabetes (yes or no), occupational activity (mostly sedentary, mostly standing, walking often, or fairly active), and walking for commuting to and from work (<20, 20 to <40, or ≥40 min of walking)

^d^Adjusted for factors in model 2 plus body mass index (kg/m^2^, continuous)

Table 3 shows the HRs of incident cases of diabetes associated with exercise intensity according to the dose of leisure-time exercise. Vigorous-intensity exercise alone or vigorous-intensity exercise combined with moderate-intensity exercise yielded greater health benefits in terms of risk reduction for incident diabetes compared with moderate-intensity exercise alone at the same dose of activity. It should be noted that not all of the reductions were statistically significant. The sensitivity analysis showed similar results in which 3,457 workers with short follow-up terms (<3 years) were excluded (data not shown).

**Table 3:** Risk of type 2 diabetes according to the dose and intensity of leisure-time exercise^a^

	Inactive		Low dose		Physical activity meeting recommendation			
	Cases (*n*)/Person-years	HR	Cases (*n*)/Person-years	HR	Median dose		High dose	
				(95 % CI)				
					Cases (*n*)/	HR	Cases (*n*)/Person-years	HR
					Person-years	(95 % CI)		(95 % CI)
Model 1^b^								
	1,210/91,266	1						
MPA alone			219/17,996	0.89	128/8,514	1.03	73/5,594	0.85
				(0.77, 1.02)		(0.86, 1.23)		(0.67, 1.08)
VPA alone			20/2,984	0.59	18/1,947	0.77	23/2,267	0.76
				(0.38, 0.92)		(0.48, 1.22)		(0.51, 1.15)
Both			18/2,099	0.66	20/2,817	0.53	41/4,254	0.68
				(0.41, 1.05)		(0.34, 0.82)		(0.50, 0.92)
Model 2^c^								
		1						
MPA alone				0.94		1.07		0.90
				(0.81, 1.08)		(0.89, 1.28)		(0.71, 1.14)
VPA alone				0.68		0.86		0.88
				(0.44, 1.06)		(0.54, 1.38)		(0.58, 1.33)
Both				0.70		0.57		0.75
				(0.44, 1.11)		(0.37, 0.89)		(0.55, 1.03)
Model 3^d^								
		1						
MPA alone				0.94		1.07		0.90
				(0.81, 1.08)		(0.88, 1.30)		(0.67, 1.21)
VPA alone				0.68		0.86		0.89
				(0.44, 1.06)		(0.54, 1.34)		(0.56, 1.41)
Both				0.70		0.57		0.76
				(0.44, 1.11)		(0.37, 0.90)		(0.52, 1.11)

Abbreviations: MPA, moderate-intensity physical activity; VPA, vigorous-intensity physical activity

^a^Medium dose of leisure-time exercise were 3.5, 10.4, and 21.0 for >0 to <7.5, 7.5 to 15.5, and ≥15.5 MET-hours per week of leisure-time exercise among those who engaged in moderate-intensity exercise alone, 3.8, 10.7, and 25.6 for >0 to <7.5, 7.5 to 15.5, and ≥15.5 MET-hours per week of leisure-time exercise among those who engaged in vigorous-intensity exercise alone, and 5.0, 10.8, and 25.7 for >0 to <7.5, 7.5 to 15.5, and ≥15.5 MET-hours per week of leisure-time exercise among those who engaged in both moderate- and vigorous-intensity exercise

^b^Adjusted for age (years, continuous) and sex

^c^Adjusted for age (years, continuous), sex, shift work (yes or no), sleep duration (<5, <6, 6 to <7, or ≥7 h per day), alcohol consumption (non-drinker, current drinker consuming <1, 1 to <2, or ≥2 go of Japanese sake equivalent per day [1 go of Japanese sake contains approximately 23 g of ethanol]), smoking (never, past, current smoker consuming 1 to 20 or ≥21 cigarettes per day), hypertension (yes or no), a family history of diabetes (yes or no), occupational activity (mostly sedentary, mostly standing, walking often, or fairly active), and walking for commuting to and from work (<20, 20 to <40, or ≥40 min of walking)

^d^Adjusted for factors in model 2 plus the total dose of moderate to vigorous-intensity physical activity during leisure time (MET-hour per week, continuous)

When we calculated the risk of diabetes associated the dose of moderate-intensity exercise, the multivariable-adjusted HRs (95 % CIs) were 1.00 (reference), 0.91 (0.80, 1.28), 1.08 (0.91, 1.28), and 0.90 (0.72, 1.13) with increasing dose of moderate-intensity exercise without BMI adjustment (Additional file 1: Table S1). The corresponding risk of diabetes associated with the dose of vigorous-intensity exercise were 1.00 (reference), 0.67 (0.51, 0.88). 0.81 (0.58, 1.13), and 0.83 (0.62, 1.11) (P for trend = 0.063) with increasing dose of vigorous-intensity exercise as shown in Additional file 1: Table S1.

Of 18 items of exercise during leisure, approximately 20 to 50 % risk reduction was observed for jogging, aerobics, tennis, soccer, and swimming, although most of the reductions did not reach statistical significance (Additional file 1: Table S2).

Occupational physical activity and walking for commuting to and from work were not associated with risk of type 2 diabetes in any models (Table 4).

**Table 4:** Association of occupational and commuting physical activity with risk of type 2 diabetes

	Cases/Subjects	Person-years	Model 1^a^	Model 2^b^	Model 3^c^
Work					
Sedentary	1,063/15,930	84,271	1.00 (reference)	1.00 (reference)	1.00 (reference)
Standing	256/3,768	19,079	1.14 (0.99, 1.30)	1.10 (0.95, 1.07)	1.13 (0.98, 1.31)
Walking	309/4,764	24,970	0.97 (0.85, 1.10)	0.94 (0.82, 1.07)	1.02 (0.89, 1.16)
Active	142/2,166	11,419	1.06 (0.89, 1.26)	1.03 (0.86, 1.24)	1.16 (0.96, 1.39)
		*P* for trend	0.77	0.71	0.23
Walking to and from work					
<20 min	901/14,383	75,443	1.00 (reference)	1.00 (reference)	1.00 (reference)
20 to <40 min	600/8,583	45,035	1.05 (0.95, 1.17)	1.06 (0.96, 1.18)	1.06 (0.96, 1.18)
≥40 min	269/3,662	19,261	1.04 (0.91, 1.20)	1.05 (0.92, 1.21)	1.07 (0.93, 1.23)
		*P* for trend	0.39	0.31	0.22

^a^Adjusted for age (years, continuous) and sex

^b^Adjusted for age (years, continuous), sex, shift work (yes or no), sleep duration (<5, <6, 6 to <7, or ≥7 h per day), alcohol consumption (non-drinker, current drinker consuming <1, 1 to <2, or ≥2 go of Japanese sake equivalent per day [1 go of Japanese sake contains approximately 23 g of ethanol]), smoking (never, past, current smoker consuming 1 to 20 or ≥21 cigarettes per day), hypertension (yes or no), a family history of diabetes (yes or no), occupational activity (mostly sedentary, mostly standing, walking often, or fairly active), walking for commuting to and from work (<20, 20 to <40, or ≥40 min of walking), and moderate-intensity exercise for vigorous-intensity exercise and vigorous-intensity exercise for moderate-intensity exercise

^c^ Adjusted for factors in model 2 plus body mass index (kg/m^2^, continuous)

## Discussion

In this large study of Japanese workers, engagement in vigorous-intensity exercise alone or vigorous-intensity exercise combined with moderate-intensity exercise was associated with a reduction in the risk of type 2 diabetes not only for doses above the recommended dose of physical activity but also for doses below the recommended level. In contrast, moderate-intensity exercise alone was not associated with a risk of type 2 diabetes even above the recommended dose of physical activity. This is the first study to compare the diabetes risk between individuals who engaged in vigorous-intensity exercise alone and individuals who engaged in moderate-intensity exercise alone. This is also among a few studies examining the relationship between occupational and commuting physical activity with diabetes.

A recent meta-analysis of cohort studies [9] suggested that risk reduction of diabetes is greater for vigorous-intensity activity compared with moderate-intensity activity. Our results of moderate-intensity and vigorous-intensity exercise dose (Additional file 1: Table S1) agree with the results of meta-analysis. However, this type of grouping used in the previous studies [9] has a limitation because individuals who engaged in moderate-intensity exercise might also perform vigorous-intensity exercise. To minimize the possibility of mixed results, we assessed the risk among individuals who engaged in moderate-intensity exercise alone or those who engaged in vigorous-intensity exercise alone (Table 3). Then, diabetes risk associated with vigorous-intensity exercise alone tended to decrease whereas that associated with moderate-intensity exercise alone did not. Our results indicate that engagement in vigorous-intensity but not moderate-intensity exercise can reduce the risk of type 2 diabetes in Asian individuals.

Although it has been suggested that any increase in the dose of physical activity from a sedentary level can reduce the risk of type 2 diabetes [7], only two US cohort studies addressed this issue. In the two studies, Japanese American men [13] and Black women [16] who engaged in <60 min of vigorous-intensity exercise per week had a slightly lower risk of diabetes (6 % and 10 %, respectively) compared with individuals who did not engage in any vigorous-intensity exercise. Again, these findings are limited due to the potential mixing of activities with different intensities. In the present study, engagement in vigorous-intensity exercise alone and both moderate- and vigorous-intensity exercise below the recommended dose (7.5 MET-hours per week) had an approximately 30 % lower risk of diabetes compared with no exercise. Although the definition of vigorous exercise and the magnitude of association differ among studies, our results together with previous findings [13, 16] support the view that vigorous-intensity exercise can reduce the risk of type 2 diabetes even when individuals engage in vigorous-intensity exercise below the recommended dose. Further studies are needed to identify the minimum amount of vigorous-intensity exercise for type 2 diabetes prevention.

In the present study among Japanese workers, moderate-intensity exercise alone was not associated with a lower risk for type 2 diabetes at even high doses. This finding disagrees with a meta-analysis of cohort studies [9], which were mainly performed in Caucasians, that demonstrated a significant inverse association between moderate physical activity and the risk of type 2 diabetes. However, as mentioned above, the approach for classification used in the previous studies has a limitation. This may partly explain the differences between ours and previous finding. In addition, cohort studies among Japanese [19] and Japanese American individuals [13] did not detect inverse associations for moderate-intensity activity. Given the limited evidence in Asians on the association between moderate-intensity exercise and the risk of type 2 diabetes [13, 19] and a finding of decreased HbA_1c_ level after moderate-intensity exercise among normal weight and obese Japanese women [30], which may be inconsistent from ours in that moderate-intensity exercise may prevent type 2 diabetes, further investigations are needed in this population.

Of the 18 items of leisure-time exercise, jogging, aerobics, tennis, soccer, and swimming were associated with a large risk reduction. In addition to these activities, we recently reported a significant risk reduction associated with strength training in detail from the same population [31]. Caution should be exercised in the interpretation of these findings because small proportion (<10 %) of workers engaged in each of activity, leading to unstable point estimates with wide confidence intervals.

We found that occupational physical activity was not associated with diabetes. This null finding is supported by three studies among Taiwanese [32], Chinese [20], and Japanese American [13]. In contrast, a Finnish study [33] reported a significant risk reduction associated with high occupational physical activity. In addition, a Japanese study [34] showed an inverse association of occupational physical activity with impaired glucose status, although the reduction was not statistically significant. We do not have a plausible explanation for inconsistent finding, however, differences in work load or socioeconomic status across the studies may partly explain.

The mechanisms of differential effects of physical activity according to the intensity level on the risk of type 2 diabetes remain unclear, but there are some possible explanations. Intervention studies among young women [35] and elderly adults [36] showed that high-intensity exercise reduced more visceral fat than moderate-intensity exercise. An intervention study among obese adults showed that the reduction in visceral fat through lifestyle modification was followed by increases in the levels of adiponectin [37], which is a protein that improves insulin sensitivity [38]. In another study among obese adults [39], a single session of high-intensity exercise showed a greater reduction in the postprandial glucose level than moderate-intensity exercise. Vigorous-intensity exercise may be effective for maintaining normal glucose metabolism for East Asians, who have a higher postprandial glucose level compared to Caucasians [40–42].

The present study has some strengths, including a large dataset of workers from a large company in Japan and the annual follow-up assessments with data on blood glucose and HbA_1c_. In most previous studies, incident cases were identified based on self-reports [11, 16, 20, 23, 43], which resulted in the under-detection of incident cases. There are several limitations in our study. First, the physical activity questionnaire used in this study was not validated. Nonetheless, the activity questionnaire in the present study is similar to the questionnaire for leisure-time physical activity used in the Shanghai Women’s Health Study [20] in which exercisers were asked to report details for up to three types of exercises/sports (i.e., type, hours/week, and years of participation in each activity) [20]. A validation study for the activity questionnaire in China [44] reported that energy expenditure of exercise estimated by the questionnaire was highly correlated with the energy expenditure of the physical activity log (*r* = 0.74). Second, we only used the physical activity information collected at baseline. The subjects may have changed their physical activity during the follow-up period. Nevertheless, such changes in physical activity would be non-differential and lead to an underestimation of the inverse association with diabetes risk. Third, unmeasured confounders (i.e., sitting time and diet) and residual confounders might have influenced the association. Fourth, the participants in the present study are young and middle-aged workers from a large company in Japan. Therefore, caution should be taken when generalizing our findings for a population with a different background.

## Conclusion

The present study among Japanese workers showed that vigorous-intensity exercise alone and vigorous-intensity exercise combined with moderate-intensity exercise were associated with a lower risk of developing type 2 diabetes, whereas moderate-intensity exercise alone was not. These results suggest that engagement in mixture of moderate- and vigorous-intensity exercise or vigorous-intensity exercise alone may help to prevent type 2 diabetes among Japanese workers.

## Additional file

Additional file 1:
**Table S1.** Association between moderate-intensity and vigorous-intensity exercise during leisure and risk of type 2 diabetes. **Table S2.** Risk of type 2 diabetes associated with specific type of leisure-time exercise. (DOCX 47 kb)

## Abbreviations

CI: confidence interval
HR: hazard ratio
IQR: interquartile range
J-ECOH: Japan Epidemiology Collaboration on Occupational Health
LTPA: leisure-time physical activity
MET: metabolic equivalent
MPA: moderate-intensity physical activity
VPA: vigorous-intensity physical activity
