# Supplementary material for: Leisure-time, occupational, and commuting physical activity and risk of type 2 diabetes in Japanese workers: a cohort study
Source: BMC Public Health. 2015 Oct 2;15:1004. doi: 10.1186/s12889-015-2362-5 (PMC4591712; doi:10.1186/s12889-015-2362-5)
Supplement: Additional file 1: — Table S1. Association between moderate-intensity and vigorous-intensity exercise during leisure and risk of type 2 diabetes. Table S2. Risk of type 2 diabetes associated with specific type of leisure-time exercise. (DOCX 47 kb) [file 12889_2015_2362_MOESM1_ESM.docx]

Table 1. Baseline characteristics according to the leisure-time exercise dose

|  | Inactive | Low dose | Physical activity meeting recommendation* | | P for trend† |
| --- | --- | --- | --- | --- | --- |
|  |  |  | Medium dose | High dose |  |
| *n* | 17,437 (65.5) | 4,331 (16.3) | 2,537 (9.5) | 2,323 (8.7) |  |
| Male, % | 14,905 (85.5) | 3,886 (89.7) | 2,281 (89.9) | 2,135 (91.9) | <0.001 |
| Age, years | 45.2 ± 8.2 | 44.6 ± 8.7 | 45.8 ± 8.5 | 46.4 ± 8.8 | <0.001 |
| BMI, kg/m^2^ | 23.3 ± 3.2 | 23.4 ± 3.2 | 23.7 ± 3.0 | 23.5 ± 2.9 | <0.001 |
| BMI ≥25 kg/m^2^ | 4,727 (27.1) | 1,173 (27.1) | 752 (29.6) | 608 (26.2) | 0.90 |
| Shift work | 3,329 (19.1) | 874 (20.2) | 415 (16.3) | 379 (16.3) | <0.001 |
| Walking <20 min to and from work | 9,299 (53.3) | 2,374 (54.8) | 1,402 (55.3) | 1,308 (56.3) | 0.002 |
| Sedentary work | 10,311 (59.1) | 2,574 (59.4) | 1,632 (64.3) | 1,413 (60.8) | 0.001 |
| Exercise dose, weekly MET-hr | 0 | 3.7 (2.2, 5.4) | 10.5 (9.0, 12.6) | 23.0 (18.2, 32.9) |  |
| Sleeping <6 hrs per day | 9,224 (52.9) | 2,021 (46.7) | 1,214 (47.9) | 1,052 (45.3) | <0.001 |
| Current drinker‡ | 1,764 (10.1) | 370 (8.5) | 250 (9.9) | 255 (11.0) | 0.31 |
| Current smoker | 7,750 (44.5) | 1,751 (40.4) | 945 (37.3) | 829 (35.7) | <0.001 |
| Hypertension | 3,092 (17.7) | 706 (16.3) | 486 (19.2) | 411 (17.7) | 0.55 |
| Family history of diabetes | 2,417 (13.9) | 570 (13.2) | 362 (14.3) | 339 (14.6) | 0.29 |

Data are shown in mean ± standard deviation for continuous variables, median (interquartile range) for exercise dose, and *n* (%) for categorical variables.

* ≥7.5 MET-hr per week.

† P for the trend was calculated using linear regression for continuous variables and logistic regression for categorical variables.

‡ Consuming ≥2 go of Japanese sake equivalent per day (1 go of Japanese sake contains approximately 23 g of ethanol).

Table 2. Hazard ratios (95% confidence intervals) of incident type 2 diabetes according to the dose of leisure-time moderate- and vigorous-intensity exercise

|  | Inactive | Low dose | Physical activity meeting recommendation | | | P for trend* |
| --- | --- | --- | --- | --- | --- | --- |
|  |  |  | Medium dose |  | High dose |  |
| Medium exercise dose | 0 | 3.7 | 10.5 |  | 23.0 |  |
| No. of subjects | 17,437 | 4,331 | 2,831 |  | 2,029 |  |
| No. of cases | 1,210 | 257 | 166 |  | 137 |  |
| Person-years | 91,266 | 23,080 | 13,278 |  | 12,116 |  |
| Cases/ 10,000 person-years | 133 | 111 | 125 |  | 113 |  |
| Model 1† | 1.00 (reference) | 0.83 (0.73, 0.95) | 0.89 (0.76, 1.05) |  | 0.78 (0.65, 0.93) | 0.003 |
| Model 2‡ | 1.00 (reference) | 0.89 (0.78, 1.02) | 0.95 (0.80, 1.11) |  | 0.85 (0.71, 1.01) | 0.06 |
| Model 3§ | 1.00 (reference) | 0.87 (0.76, 1.00) | 0.92 (0.78, 1.08) |  | 0.83 (0.69, 0.99) | 0.024 |

* P for the trend was calculated using Cox proportional hazard regression, and ordinal numbers 0, 4, 11, and 23 were assigned to increasing levels of leisure-time physical activity, which was treated as continuous variable.

† Adjusted for age (years, continuous) and sex.

‡ Adjusted for age (years, continuous), sex, shift work (yes or no), sleep duration (<5, <6, 6 to <7, or ≥7 hours per day), alcohol consumption (non-drinker, current drinker consuming <1, 1 to <2, or ≥2 go of Japanese sake equivalent per day [1 go of Japanese sake contains approximately 23 g of ethanol]), smoking (never, past, current smoker consuming 1 to 20 or ≥21 cigarettes per day), hypertension (yes or no), a family history of diabetes (yes or no), occupational activity (mostly sedentary, mostly standing, walking often, or fairly active), and walking for commuting to and from work (<20, 20 to <40, or ≥40 min of walking).

§ Adjusted for factors in model 2 plus body mass index (kg/m^2^, continuous).

Table 3. Risk of type 2 diabetes according to the dose and intensity of leisure-time exercise*

|  | Inactive |  |  | Low dose | |  | Physical activity meeting recommendation | | | | |
| --- | --- | --- | --- | --- | --- | --- | --- | --- | --- | --- | --- |
|  | Cases (n)/ Person-years | HR |  | Cases (n)/ Person-years | HR  (95% CI) |  | Median dose | |  | High dose | |
|  |  |  |  |  |  |  | Cases (n)/  Person-years | HR  (95% CI) |  | Cases (n)/ Person-years | HR  (95% CI) |
| Model 1† |  |  |  |  |  |  |  |  |  |  |  |
|  | 1,210/91,266 | 1 |  |  |  |  |  |  |  |  |  |
| MPA alone |  |  |  | 219/17,996 | 0.89  (0.77, 1.02) |  | 128/8,514 | 1.03  (0.86, 1.23) |  | 73/5,594 | 0.85  (0.67, 1.08) |
| VPA alone |  |  |  | 20/2,984 | 0.59  (0.38, 0.92) |  | 18/1,947 | 0.77  (0.48, 1.22) |  | 23/2,267 | 0.76  (0.51, 1.15) |
| Both |  |  |  | 18/2,099 | 0.66  (0.41, 1.05) |  | 20/2,817 | 0.53  (0.34, 0.82) |  | 41/4,254 | 0.68  (0.50, 0.92) |
| Model 2‡ |  |  |  |  |  |  |  |  |  |  |  |
|  |  | 1 |  |  |  |  |  |  |  |  |  |
| MPA alone |  |  |  |  | 0.94  (0.81, 1.08) |  |  | 1.07  (0.89, 1.28) |  |  | 0.90  (0.71, 1.14) |
| VPA alone |  |  |  |  | 0.68  (0.44, 1.06) |  |  | 0.86  (0.54, 1.38) |  |  | 0.88  (0.58, 1.33) |
| Both |  |  |  |  | 0.70  (0.44, 1.11) |  |  | 0.57  (0.37, 0.89) |  |  | 0.75  (0.55, 1.03) |
| Model 3§ |  |  |  |  |  |  |  |  |  |  |  |
|  |  | 1 |  |  |  |  |  |  |  |  |  |
| MPA alone |  |  |  |  | 0.94  (0.81, 1.08) |  |  | 1.07  (0.88, 1.30) |  |  | 0.90  (0.67, 1.21) |
| VPA alone |  |  |  |  | 0.68  (0.44, 1.06) |  |  | 0.86  (0.54, 1.34) |  |  | 0.89  (0.56, 1.41) |
| Both |  |  |  |  | 0.70  (0.44, 1.11) |  |  | 0.57  (0.37, 0.90) |  |  | 0.76  (0.52, 1.11) |

Abbreviations: MPA, moderate-intensity physical activity; VPA, vigorous-intensity physical activity.

*Medium dose of leisure-time exercise were 3.5, 10.4, and 21.0 for >0 to <7.5, 7.5 to 15.5, and ≥15.5 MET-hours per week of leisure-time exercise among those who engaged in moderate-intensity exercise alone, 3.8, 10.7, and 25.6 for >0 to <7.5, 7.5 to 15.5, and ≥15.5 MET-hours per week of leisure-time exercise among those who engaged in vigorous-intensity exercise alone, and 5.0, 10.8, and 25.7 for >0 to <7.5, 7.5 to 15.5, and ≥15.5 MET-hours per week of leisure-time exercise among those who engaged in both moderate- and vigorous-intensity exercise.

† Adjusted for age (years, continuous) and sex.

‡ Adjusted for age (years, continuous), sex, shift work (yes or no), sleep duration (<5, <6, 6 to <7, or ≥7 hours per day), alcohol consumption (non-drinker, current drinker consuming <1, 1 to <2, or ≥2 go of Japanese sake equivalent per day [1 go of Japanese sake contains approximately 23 g of ethanol]), smoking (never, past, current smoker consuming 1 to 20 or ≥21 cigarettes per day), hypertension (yes or no), a family history of diabetes (yes or no), occupational activity (mostly sedentary, mostly standing, walking often, or fairly active), and walking for commuting to and from work (<20, 20 to <40, or ≥40 min of walking).

§ Adjusted for factors in model 2 plus the total dose of moderate to vigorous-intensity physical activity during leisure time (MET-hour per week, continuous).

Table 4. Association of occupational and commuting physical activity with risk of type 2 diabetes

|  | Cases/Subjects | Person-years | Model 1 | Model 2 | Model 3 |
| --- | --- | --- | --- | --- | --- |
| Work |  |  |  |  |  |
| Sedentary | 1,063/15,930 | 84,271 | 1.00 (reference) | 1.00 (reference) | 1.00 (reference) |
| Standing | 256/3,768 | 19,079 | 1.14 (0.99, 1.30) | 1.10 (0.95, 1.07) | 1.13 (0.98, 1.31) |
| Walking | 309/4,764 | 24,970 | 0.97 (0.85, 1.10) | 0.94 (0.82, 1.07) | 1.02 (0.89, 1.16) |
| Active | 142/2,166 | 11,419 | 1.06 (0.89, 1.26) | 1.03 (0.86, 1.24) | 1.16 (0.96, 1.39) |
|  |  | P for trend | 0.77 | 0.71 | 0.23 |
| Walking to and from work |  |  |  |  |  |
| <20 min | 901/14,383 | 75,443 | 1.00 (reference) | 1.00 (reference) | 1.00 (reference) |
| 20 to <40 min | 600/8,583 | 45,035 | 1.05 (0.95, 1.17) | 1.06 (0.96, 1.18) | 1.06 (0.96, 1.18) |
| ≥40 min | 269/3,662 | 19,261 | 1.04 (0.91, 1.20) | 1.05 (0.92, 1.21) | 1.07 (0.93, 1.23) |
|  |  | P for trend | 0.39 | 0.31 | 0.22 |

† Adjusted for age (years, continuous) and sex.

‡ Adjusted for age (years, continuous), sex, shift work (yes or no), sleep duration (<5, <6, 6 to <7, or ≥7 hours per day), alcohol consumption (non-drinker, current drinker consuming <1, 1 to <2, or ≥2 go of Japanese sake equivalent per day [1 go of Japanese sake contains approximately 23 g of ethanol]), smoking (never, past, current smoker consuming 1 to 20 or ≥21 cigarettes per day), hypertension (yes or no), a family history of diabetes (yes or no), occupational activity (mostly sedentary, mostly standing, walking often, or fairly active), walking for commuting to and from work (<20, 20 to <40, or ≥40 min of walking), and moderate-intensity exercise for vigorous-intensity exercise and vigorous-intensity exercise for moderate-intensity exercise.

§ Adjusted for factors in model 2 plus body mass index (kg/m^2^, continuous).

Supplementary table 1. Association between moderate-intensity and vigorous-intensity exercise during leisure and risk of type 2 diabetes

|  | Inactive | Low dose | Physical activity meeting recommendation | | | P for trend |
| --- | --- | --- | --- | --- | --- | --- |
|  |  |  | Medium dose |  | High dose |  |
| Moderate-intensity |  |  |  |  |  |  |
| Moderate-intensity exercise dose, medium | 0 | 3.4 | 10.3 |  | 20.8 |  |
| No. of subjects | 18,747 | 4,613 | 2,002 |  | 1,266 |  |
| No. of cases | 1,271 | 267 | 150 |  | 82 |  |
| Person-years | 98,464 | 24,439 | 10,349 |  | 6,488 |  |
| Cases/ 10,000 person-years | 129 | 109 | 145 |  | 126 |  |
| Model 1* | 1.00 (reference) | 0.82 (0.72, 0.93) | 1.01 (0.85, 1.19) |  | 0.85 (0.68, 1.06) | 0.16 |
| Model 2† | 1.00 (reference) | 0.91 (0.80, 1.28) | 1.08 (0.91, 1.28) |  | 0.90 (0.72, 1.13) | 0.64 |
| Model 3‡ | 1.00 (reference) | 0.89 (0.77, 1.02) | 1.01 (0.85, 1.20) |  | 0.82 (0.66, 1.03) | 0.13 |
| Vigorous-intensity |  |  |  |  |  |  |
| Vigorous-intensity exercise dose, medium | 0 | 3.4 | 10.7 |  | 25.0 |  |
| No. of subjects | 23,584 | 1,407 | 761 |  | 876 |  |
| No. of cases | 1,630 | 56 | 37 |  | 47 |  |
| Person-years | 123,370 | 7,626 | 4,076 |  | 4,667 |  |
| Cases/ 10,000 person-years | 132 | 73 | 91 |  | 101 |  |
| Model 1* | 1.00 (reference) | 0.60 (0.46, 0.78) | 0.73 (0.53, 1.01) |  | 0.74 (0.55, 0.99) | 0.002 |
| Model 2† | 1.00 (reference) | 0.67 (0.51, 0.88) | 0.81 (0.58, 1.13) |  | 0.83 (0.62, 1.11) | 0.063 |
| Model 3‡ | 1.00 (reference) | 0.70 (0.53, 0.92) | 0.86 (0.62, 1.19) |  | 0.94 (0.70, 1.26) | 0.32 |

* Adjusted for age (years, continuous) and sex.

†Adjusted for age (years, continuous), sex, shift work (yes or no), sleep duration (<5, <6, 6 to <7, or ≥7 hours per day), alcohol consumption (non-drinker, current drinker consuming <1, 1 to <2, or ≥2 go of Japanese sake equivalent per day [1 go of Japanese sake contains approximately 23 g of ethanol]), smoking (never, past, current smoker consuming 1 to 20 or ≥21 cigarettes per day), hypertension (yes or no), a family history of diabetes (yes or no), occupational activity (mostly sedentary, mostly standing, walking often, or fairly active), walking for commuting to and from work (<20, 20 to <40, or ≥40 min of walking), and mutually adjusted for different intensity of exercise during leisure (i.e., adjustment for moderate-intensity exercise dose [0, >0 to <7.5, 7.5 to <15.5, or 15.5 MET-hours per week] for vigorous-intensity exercise and vigorous-intensity exercise for moderate-intensity exercise).

‡Adjusted for factors in model 2 plus body mass index (kg/m^2^, continuous).

Supplementary table 2. Risk of type 2 diabetes associated with specific type of leisure-time exercise

|  |  |  | Hazard ratios (95% confidence intervals) | | |
| --- | --- | --- | --- | --- | --- |
| Type of activity | Cases/No. of subjects |  | Model 1* | Model 2† | Model 3‡ |
| Walking§ |  |  |  |  |  |
| No | 1,582/24,163 |  | 1.00 (reference) | 1.00 (reference) | 1.00 (reference) |
| Yes | 188/2,465 |  | 1.03 (0.88, 1.20) | 1.05 (0.90, 1.22) | 0.99 (0.85, 1.15) |
| Walking fast |  |  |  |  |  |
| No | 1,714/25,932 |  | 1.00 (reference) | 1.00 (reference) | 1.00 (reference) |
| Yes | 56/696 |  | 1.05 (0.80, 1.37) | 1.07 (0.82, 1.40) | 1.10 (0.84, 1.44) |
| Light jogging |  |  |  |  |  |
| No | 1,718/25,675 |  | 1.00 (reference) | 1.00 (reference) | 1.00 (reference) |
| Yes | 52/953 |  | 0.78 (0.59, 1.03) | 0.88 (0.67, 1.16) | 0.91 (0.69, 1.20) |
| Jogging |  |  |  |  |  |
| No | 1,726/25,745 |  | 1.00 (reference) | 1.00 (reference) | 1.00 (reference) |
| Yes | 44/883 |  | 0.68 (0.51, 0.92) | 0.75 (0.56, 1.02) | 0.85 (0.63, 1.15) |
| Bicycle |  |  |  |  |  |
| No | 1,740/26,112 |  | 1.00 (reference) | 1.00 (reference) | 1.00 (reference) |
| Yes | 30/516 |  | 0.92 (0.64, 1.32) | 0.92 (0.64, 1.32) | 0.94 (0.65, 1.34) |
| Swimming |  |  |  |  |  |
| No | 1,744/25,972 |  | 1.00 (reference) | 1.00 (reference) | 1.00 (reference) |
| Yes | 26/656 |  | 0.60 (0.41, 0.89) | 0.65 (0.44, 0.96) | 0.70 (0.41, 1.03) |
| Aerobics |  |  |  |  |  |
| No | 1,761/26,333 |  | 1.00 (reference) | 1.00 (reference) | 1.00 (reference) |
| Yes | 9/295 |  | 0.64 (0.33, 1.22) | 0.68 (0.35, 1.30) | 0.71 (0.37, 1.38) |
| Radio gymnastics |  |  |  |  |  |
| No | 1,720/25,832 |  | 1.00 (reference) | 1.00 (reference) | 1.00 (reference) |
| Yes | 50/796 |  | 0.85 (0.64, 1.12) | 0.89 (0.67, 1.18) | 0.93 (0.70, 1.23) |
| Jump rope |  |  |  |  |  |
| No | 1,764/26,519 |  | 1.00 (reference) | 1.00 (reference) | 1.00 (reference) |
| Yes | 6/109 |  | 0.77 (0.35, 1.72) | 0.85 (0.38, 1.90) | 0.86 (0.39, 1.92) |
| Soccer |  |  |  |  |  |
| No | 1,753/26,025 |  | 1.00 (reference) | 1.00 (reference) | 1.00 (reference) |
| Yes | 17/603 |  | 0.49 (0.31, 0.80) | 0.52 (0.32, 0.83) | 0.52 (0.32, 0.85) |
| Golf practice |  |  |  |  |  |
| No | 1,586/24,023 |  | 1.00 (reference) | 1.00 (reference) | 1.00 (reference) |
| Yes | 184/2,605 |  | 0.95 (0.82, 1.11) | 1.01 (0.86, 1.17) | 0.95 (0.81, 1.10) |
| Golf |  |  |  |  |  |
| No | 1,674/25,130 |  | 1.00 (reference) | 1.00 (reference) | 1.00 (reference) |
| Yes | 96/1,498 |  | 0.85 (0.69, 1.05) | 0.88 (0.72, 1.08) | 0.81 (0.66, 0.99) |
| Baseball |  |  |  |  |  |
| No | 1,731/25,952 |  | 1.00 (reference) | 1.00 (reference) | 1.00 (reference) |
| Yes | 39/676 |  | 0.95 (0.69, 1.30) | 0.95 (0.69, 1.31) | 0.82 (0.60, 1.14) |
| Softball |  |  |  |  |  |
| No | 1,737/26,045 |  | 1.00 (reference) | 1.00 (reference) | 1.00 (reference) |
| Yes | 33/583 |  | 0.77 (0.54, 1.08) | 0.81 (0.57, 1.14) | 0.74 (0.52, 1.04) |
| Tennis |  |  |  |  |  |
| No | 1,746/26,135 |  | 1.00 (reference) | 1.00 (reference) | 1.00 (reference) |
| Yes | 24/493 |  | 0.70 (0.47, 1.04) | 0.77 (0.52, 1.16) | 0.81 (0.54, 1.21) |
| Table tennis |  |  |  |  |  |
| No | 1,764/26,540 |  | 1.00 (reference) | 1.00 (reference) | 1.00 (reference) |
| Yes | 6/88 |  | 0.99 (0.44, 2.21) | 1.05 (0.47, 2.34) | 1.12 (0.50, 2.49) |
| Pang Pong |  |  |  |  |  |
| No | 1,755/26,317 |  | 1.00 (reference) | 1.00 (reference) | 1.00 (reference) |
| Yes | 15/311 |  | 0.78 (0.47, 1.29) | 0.81 (0.49, 1.35) | 0.85 (0.51, 1.41) |
| Badminton |  |  |  |  |  |
| No | 1,758/26,402 |  | 1.00 (reference) | 1.00 (reference) | 1.00 (reference) |
| Yes | 12/226 |  | 0.91 (0.51, 1.60) | 0.95 (0.54, 1.67) | 0.94 (0.53, 1.66) |

*Adjusted for age (years, continuous) and sex.

† Further adjusted for shift work (yes or no), sleep duration (<5, <6, 6 to <7, or ≥7 hours per day), alcohol consumption (non-drinker, current drinker consuming <1, 1 to <2, or ≥2 go of Japanese sake equivalent per day [1 go of Japanese sake contains approximately 23 g of ethanol]), smoking (never, past, current smoker consuming 1 to 20 or ≥21 cigarettes per day), hypertension (yes or no), a family history of diabetes (yes or no), occupational activity (mostly sedentary, mostly standing, walking often, or fairly active), walking for commuting to and from work (<20, 20 to <40, or ≥40 min of walking).

‡ Additionally adjusted for body mass index (continuous).

§Excluding walking for commuting and work.
